# The Function of miRNA in Hepatic Cancer Stem Cell

**DOI:** 10.1155/2013/358902

**Published:** 2013-12-09

**Authors:** Wei Qi, Weicheng Liang, Huiqing Jiang, Mary Miuyee Waye

**Affiliations:** ^1^Department of Gastroenterology, The Second Hospital of Hebei Medical University, Hebei Key Laboratory of Gastroenterology, Hebei Institute of Gastroenterology, Shijiazhuang, Hebei 23000, China; ^2^School of Biomedical Sciences, The Chinese University of Hong Kong, Shatin, Hong Kong; ^3^Croucher Laboratory for Human Genomics, The Chinese University of Hong Kong, Shatin, Hong Kong

## Abstract

Hepatocellular carcinoma (HCC) is the fifth most common cancer worldwide and ranks third in the leading causes of cancer patient's death. Cancer stem cells (HSCs), also known as tumor-initiating cells, have been reported in multiple subtypes of HCC and are considered as the master regulators of HCC initiation, chemotherapy drug resistance, tumor metastasis, and progression. In spite of their clinical importance, the detailed mechanism about how HSCs are intricately regulated in the molecular level remains elusive. MicroRNA (miRNA), a class of newly emerging small noncoding RNAs, has been demonstrated to serve as a vital player in modulating a number of biological activities ranging from embryogenesis to programmed cell death as well as the maintenance of HSCs. In this review, we synthesize these latest findings of miRNA regulation of HSCs and try to elucidate their mechanistic roles in orchestrating cellular equilibrium. This recent progress underlies the functional role of miRNA in cellular transformation of liver cancer, which has largely extended our knowledge how HSCs are controlled by miRNA network, and in the development of novel miRNA-based anticancer therapies specifically targeting HSCs in the coming future.

## 1. Introduction

Twenty years ago, two small noncoding but functional RNAs, lin-4 and let-7, were first identified to control the developmental timing in the nematode *Caenorhabditis elegans *[1, 2]. Since then, extensive studies have been conducted to uncover the functional role of miRNA in multiple biological activities, ranging from embryonic development to cell death [3–7]. Moreover, compelling evidence has revealed that miRNA, the small endogenous noncoding RNA, serves as an important player in modulating diverse cellular process through targeting the protein-coding genes or even long noncoding RNAs. Hence, the discovery of microRNA (miRNA) largely extends our understanding about gene expression and regulation. It is estimated that the human genome may encode more than 1000 miRNAs and a number of miRNAs are highly conserved across a wide range of distinct species [8, 9].

## 2. The Biogenesis of miRNA

**Figure 1:**
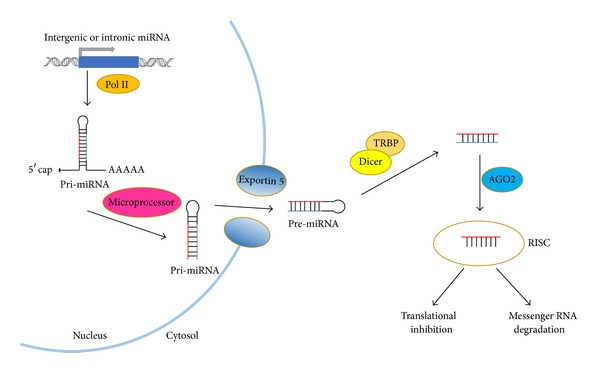
Schematic overview of canonical miRNA biogenesis. The pri-miRNA is transcribed from intergenic or intronic region and subsequently spliced by microprocessors, namely, Drosha/DGCR8 or spliceosome. On export from the nucleus to cytosol via Exportin 5, the pre-miRNA is incorporated into RISC complex after Dicer cleavage. Being unwound in RISC complex, one strand stays in the RISC complex as the mature miRNA while the other strand undergoes degradation. Binding to target messenger RNA by miRNAs in RISC complex is followed by translation inhibition and/or mRNA degradation in the cytoplasm.

The biogenesis of miRNAs includes multiple steps including transcription, nuclear processing, exportation, and cytoplasmic processing as well as translation inhibition or activation [10, 11]. miRNAs are transcribed as primary miRNAs (pri-miRNAs) by RNA polymerase II or III with 5′ end caps and 3′ end poly-A tails [12–14]. The primary miRNAs from intergenic region are processed by the protein complex including nuclease, DiGeorge syndrome critical region gene 8 (DGCR8) and Drosha, whereas the other primary miRNAs from intragenic region are processed by spliceosomes [15–17]. After modifications from the above microprocessors, the precursor miRNAs (pre-miRNAs) derived from the primary miRNAs are exported to the cytoplasm with the assistance of the protein Exportin-5 [18]. In the cytoplasm, the pre-miRNAs are cleaved and processed by the nuclease termed Dicer [13] and its partner, human immunodeficiency virus transactivating response RNA binding protein (TRBP), which produces final products around 21–23 nucleotides in length with 5′ phosphates and 2-nucleotide 3′ overhangs. Finally, two complementary small RNA fragments are generated and subsequently designated as −5p and −3p. The mature miRNAs are incorporated into the RNA-induced silencing complex (RISC) and then mediate messenger RNA degradation and/or translational inhibition (Figure 1) [10, 11].

## 3. RNA Activation

Intriguingly, emerging evidence indicates that small RNAs also act as transcriptional activators via interacting with promoter region or untranslated region [19–21]. Vasudevan et al. found that miRNAs inhibit gene translation in proliferating state but stimulate it in a quiescent state. They further demonstrated that the AGO2-miR369-3 complex serves as scaffold proteins to recruit the fragile X-related protein 1 (FXR1) and subsequently activates mRNA translation by binding to the 3′ UTR region of TNF*α* mRNA [19]. Ørom et al. unraveled that miR-10a interacts with the 5′ UTR region of multiple ribosomal protein mRNAs and plays an important role in translational induction of these mRNAs to fight against survival stress including amino acid starvation [20]. Regardless of the untranslated region, some groups also identified several small RNAs stimulating gene translation through interacting with specific promoter region [22–25]. For instance, Li et al. identified that several artificially synthesized dsRNAs or endogenous miRNA could activate gene transcription of E-Cadherin by binding to the promoter region of E-Cadherin [23, 24]. Nevertheless, the detailed mechanism underlying RNA activation remains largely unknown at the present time. Further investigations are badly needed to elucidate the molecular mechanism regarding how miRNAs activate gene expression via interacting with promoter region or untranslated region.

## 4. Regulation of Embryonic Stem Cells by miRNA

Recent studies highlight the function of miRNAs in controlling the self-renewal and pluripotency as well as differentiation of progeny cells. The overall function of miRNAs in mouse embryonic stem cells (ESCs) has been studied by generating the Dicer-null mice. The ESCs derived from Dicer-null mice display embryonic lethality as well as severe defects in differentiation both *in vitro* and* in vivo*, indicating that Dicer participates in diverse essential biological activities ranging from embryogenesis to maintaining the genome stability [26, 27]. Moreover, researchers also generated the DGCR8-deficient mouse ESCs and these mice show defects in cell proliferation and cell-cycle transition. Further experiments verified that DGCR8 is fundamental for miRNA biogenesis and that miRNAs are master regulator of embryonic cell differentiation [28].

Since Shinya Yamanaka's generating the induced pluripotent stem cells (iPS cells) first from mouse fibroblast and subsequently from human via using pluripotent stem cell transcription factor including OCT3/4, SOX2, KLF4, and MYC, plenty of methods including viral transduction or small-molecule compounds for producing iPS cells *in vitro* have been developed [29–32]. These new approaches are frequently used in combination with small molecules that serve as potent enhancers of iPS cell development [31, 32]. Currently, some miRNA clusters, highly expressed in embryonic stem cells, were identified to promote iPS cells reprogramming in conjunction with the Yamanaka factors (OCT3/4, SOX2, KLF4, and MYC) [33, 34]. Nevertheless, how these miRNAs promote iPS cells reprogramming remains elusive but may be partially related to their ability to orchestrate cell cycle transition and cell death [33]. Of the miRNAs preferentially expressed in iPS cells, the miR302/367 cluster is directly regulated by transcription factors SOX2 and OCT3/4, both of which are essential for iPS reprogramming [35]. In addition, some transcription factors involved in maintaining stem cell pluripotency, including SOX2, OCT3/4, NANOG, and TCF3, were also found to directly bind to the promoter regions of ESC-specific miRNAs [36]. On the other hand, some ESC-specific miRNAs were found to directly target the pluripotency genes at the translational level. For instance, miR-134, miR-296, and miR-470 are significantly upregulated during the differentiation of mouse embryonic stem cells after induction with retinoic-acid and target NANOG, OCT3/4, and SOX2 by binding to their coding regions, inducing the mouse embryonic stem cells morphology changes and resulting in a novel phenotype [37]. The RNA binding protein lin-28, a biomarker of undifferentiated ESCs, is a *bona fide* target for let-7 during developmental commitment [38, 39]. Conversely, further studies illustrated that the biogenesis of let-7 family is tightly modulated by lin-28. For instance, lin-28 could suppress let-7 maturation by binding to the loop of the primary let-7 [38, 40] or the stem part of precursor let-7 [39, 41]. Thus, let-7 and lin-28 form an automatic negative feedback loop to precisely modulate each other's expression level.

## 5. Hepatic Cancer Stem Cells and miRNA

Hepatocellular carcinoma (HCC), affecting more than half million individuals annually, is the fifth leading cause of cancer and ranks third in cancer mortality worldwide [42]. The majority of HCC patients are diagnosed in advanced stages with ineffective therapeutic options and unfavorable prognosis [43]. Resection and transplantation are not effective for HCC in advanced stages [44]. Furthermore, the emergence of cellular resistance to current chemotherapy and radiotherapy modalities leaves this disease with frequent occurrence of relapses [45, 46]. In order to identify novel treatment strategies and tumor markers associated with tumorigenesis of HCC, intensive efforts have been made [47].

A growing body of evidence in cancer biology has indicated that tumor tissues are organized in a hierarchy consisted of heterogeneous cell populations, and the capability to maintain tumorigenesis exclusively relies on a small population of cells called cancer stem cells (CSCs) [48]. Cancer stem cells are key players sustaining tumor formation and growth and are also endowed with stem cell properties [49, 50], including the self-renewal ability and pluripotency. Current chemotherapy and radiotherapy modalities eliminate the bulk of cancerous cells but fail to eliminate all of CSCs that maintain a high capacity for renewal [46, 51]. Similar to normal stem cells, cancer stem cells generate their progeny and have the ability to reconstitute tumors. Currently, it is reported that CSCs exist in glioblastoma, leukemia, prostate, breast, lung, gastric, and colon cancer [49, 52–57]. Similar to many other cancers, CSCs in HCC have also been proven [58].

It is postulated that CSCs derive from stem cells carrying mutations or dedifferentiated mature cells. It is believed that transformation from a normal cell to a cancerous cell requires approximately 3 to 6 genetic events [59]. As stem cells maintain longer life span compared to their progeny, it is postulated that stem cells have the highest potential to accumulate the requisite number of mutations to disturb intrinsic mechanisms regulating normal cell metabolisms and proliferation. On the other side, the dedifferentiation of mature cells possibly happens in tumorigenesis. For example, in the process of epithelial mesenchymal transition (EMT), mature cell becomes more “stem cell-like” with certain upregulated transcription factors, such as SOX2 and KLF4.

Based on specific surface markers and functional properties, hepatic CSCs have been isolated from heterogeneous tumor tissues. Various markers have been found for hepatic cancer stem cells, including CD133, CD90, and EpCAM. Depending on the surrogate characteristics, functional assays to isolate hepatic CSCs have been developed, including side population approach, ALDEFLUOR-approach, sphere formation, and asymmetric division [60, 61]. Due to the plasticity of CSCs, it is not appropriate to identify and isolate hepatic CSCs by a single marker or functional assay. It is necessary to define hepatic CSCs by an integrated approach combining proper immunogenic markers and functional assay.

Several signaling pathways have been unraveled in hepatocarcinogenesis including MET, MYC, TGF-*β*, Hedgehog, p53, WNT/*β*-Catenin, and EGF. Many of them overlap with pathways associated with hepatic progenitor cells. The functions of miRNA and other noncoding RNAs in hepatic CSCs have also been reported.

**Figure 2:**
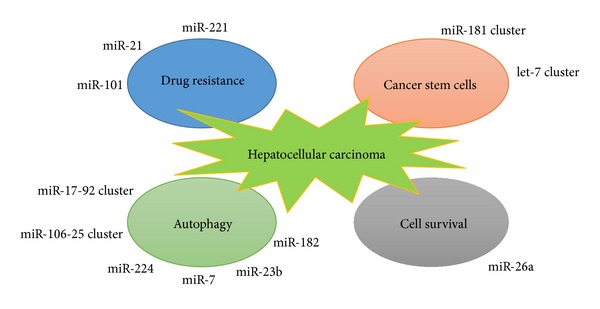
The role of miRNAs in regulating key properties of hepatic cancer cells. Several miRNAs, via targeting a variety of downstream signaling pathways, act synergistically to regulate several key biological properties of liver cancer cells including drug resistance, stemness properties, cell survival, and autophagy.

Based on the computational and experimental evidence, it has been estimated that miRNAs encoded by the human genome could modulate around 60% of mammalian genes, highlighting the importance of miRNAs in orchestrating gene expression. Furthermore, it has been reported that a single miRNA may affect the expression levels of a number of target genes [62, 63]. Together, it will not be surprising that even a single type of miRNA may enable normal cells to be transformed into cancerous cells [64–68]. Two recent studies highlight the functional role of miRNAs in regulating carcinogenesis through modulating the stemness properties of CSCs [69, 70] (Figure 2).

By detecting EpCAM and Alpha-fetoprotein (AFP) expression status in conjunction with transcriptome analysis in HCC tumor specimens, Yamashita et al. distinguished two HCC subtypes for prognostic prediction, and they are EpCAM^+^AFP^+^ HCC (referred to as hepatic stem cell-like HCC; HpSC-HCC) with enhanced metastatic properties and poor outcome and EpCAM^−^AFP^−^ HCC (referred to as mature hepatocyte-like HCC; MH-HCC) with good prognosis [71, 72]. Later, by comparing miRNA expression profiles between hepatic stem cell-like hepatocellular carcinoma (HpSC-HCC) and mature hepatocyte-like hepatocellular carcinoma (MH-HCC), Ji et al. unraveled the specific miRNAs preferentially expressed in HpSC-HCC and demonstrated that the highly conserved miR-181 family are highly expressed in EpCAM^+^AFP^+^ cells isolated from AFP^+^ HCC tumor specimens [69]. Further experiments elucidated that miR-181 family exert their function through targeting Caudal type homeobox transcription factor 2 (CDX2), GATA binding protein 6 (GATA6), and Nemo-like kinase (NLK), which are essential for hepatic cell differentiation and Wnt pathway [69].

After isolating HSCs from liver cancer tissues by using positive markers including OCT3/4, CD133, Nestin, and AFP as well as Carcino-embryonic antigen (CEA), Meng et al. evaluated the miRNA signatures in human HSCs by microarray-based approach followed by the validation with real-time PCR [70]. Among the miRNAs upregulated in HSCs, let-7 and miR-181 family are of great interest because of their vital role in regulating stemness characteristics (Figure 2). They also confirmed that these two miRNA families are regulated by IL-6 and Twist, respectively.

The sophisticated molecular alternation which occurs during hepatocarcinogenesis provides a long-lasting challenge for researchers. To address this issue, numerous investigations have been conducted. However, although encouraging progress elucidating molecular mechanism on miRNA and HSCs has been achieved, there is a substantial need for more thorough investigations on how miRNAs are involved in HSCs development and tumor progression.

## 6. miRNA as a Therapeutic Tool in HCC: From Bench to Bedside

Currently, the discovery of aberrant miRNA expression profiles in liver cancer largely extends our understanding of HCC and recent studies have shown that miRNA emerges as a promising tool with higher specificity and accuracy for clinical diagnosis and prognosis [73].

Compelling evidences have implied the potential application of miRNA as a novel strategy in cancer therapy for HCC. At present, to develop the potential therapeutic tools for caner therapy, researchers utilize viral vector system to elevate tumor suppressive miRNAs that eliminate carcinogenesis or anti-miRNA oligonucleotides (to repress the oncogenic miRNAs) which promotes tumorigenesis [11].

Among the established approaches for the *in vivo* miRNA delivery, the adenoassociated viral vectors were found to be a promising therapeutic strategy for cancer therapy because of the lower risk of vector-related toxicities as well as higher gene transfer efficacy [74, 75]. Kota et al. found out that the expression level of miR-26a is significantly downregulated in the MYC-induced hepatocarcinoma murine model (tet-o-MYC; LAP-tTA mice), and this result was further confirmed by detecting the expression profiling of miR-26a in human HCC and normal liver biopsies [75]. Subsequent experiments showed that the tumor suppressive role of miR-26a may be related to their ability to regulate the cell cycle progression via targeting Cyclin D2 and Cyclin E2, two influential players in G1/S cell phase transition. To further characterize the function of miR-26a* in vivo *and *in vitro*, they utilized an adenoassociated virus carrying miRNA-26a gene to infect HepG2 cells and mouse model with MYC-induced hepatocarcinoma and found out that ectopic expression of miR-26a results in blockage of cell proliferation and induction of tumor cell apoptosis, suggesting that delivery of miRNAs with tumor suppressive function may provide a novel strategy to develop miRNA therapy.

Besides viral vectors, artificially synthesized miRNA or anti-miRNA oligonucleotides were also shown to serve as an important therapeutic strategy for cancer therapy. Park et al. evaluated the therapeutic efficacy of anti-miR-221 oligonucleotides with different chemical modification forms [76]. Of 9 modification forms evaluated, a cholesterol-modified isoform of anti-miR-221 oligonucleotides significantly impairs *in vitro *and* in vivo* cancer cell proliferation, indicating that this target agent may benefit anticancer treatments for HCC patients [76]. In terms of the important biological implication of miR-221 in HCC, Callegari et al. generated a transgenic mouse model with inappropriate overexpression of miR-221 in the liver, which spontaneously develops liver tumors in a number of mice [77]. Further experiments showed that *in vivo* delivery of antisense 2′-*O*-methyloligoribonucleotide targeting miR-221 results in a prolonged survival and significant reduction of the number of tumor nodules in murine HCC models [77]. Similarly, restoration of some miRNAs which serve as tumor suppressors could significantly block tumorigenesis and metastasis* in vivo* [78–80]. It is well characterized that Osteopontin (OPN) is overexpressed in liver cancer patients with enhanced metastasis and poor prognosis, and repression of OPN using neutralizing antibody could significantly weaken cell migration and invasiveness *in vitro* [81]. The artificial miRNAs, designated for targeting OPN, could significantly inhibit OPN expression in HCCLM3 cell line and result in decreased *in vivo *tumor growth and lung metastasis through the repression of matrix metalloproteinase 2 (MMP2) and NF-*κ*B pathways [82].

Furthermore, miRNAs are shown to reduce drug sensitivity of tumor cells to chemotherapy. Hepatoma cells with elevated expression of miR-21 were found to lower drug sensitivity to the cellular cytotoxicity induced by Interferon-*α* (IFN-*α*) in conjunction with 5-Fluorouracil (5-FU), and high level of miR-21 in clinical human hepatoma biopsies indicates unfavorable response to the IFN-*α*/5-FU combination chemotherapy [83]. Furthermore, ectopic expression of anti-miR-21 oligonucleotides renders HCC cells sensitive to IFN-*α*/5-FU, and similar results can be achieved by transfection of siRNAs against phosphatase and tensin homologue (PTEN) or programmed cell death 4 (PDCD4), two *bona fide *targets for miRN-21 [83]. MiR-101, a frequently downregulated miRNA in HCC, sensitizes HepG2 cells to apoptosis induced by serum starvation or chemotherapeutic drugs through targeting myeloid cell leukemia sequence 1 (Mcl-1), a well-characterized antiapoptotic member of Bcl-2 family [84].

All together, these novel findings suggest that patients may benefit from specific miRNA adjuvant administration based on diverse miRNA expression profiles in different individuals suffering from HCC.

## 7. Role of Autophagy and miRNA in Liver Cancer

Autophagy is a tightly regulated cellular catabolic process involving the clearance of organelles and macromolecules. This genetically programmed process includes serial steps ranging from initiation of the phagophore assembly site to the formation of double membrane vesicle complex termed autophagosome, which encapsulates cytoplasmic organelles and proteins and subsequently fuses with lysosomes for the degradation of these intracellular constituents [85]. As a constantly changing process, autophagy provides an alternative energy source for the adaptation of immediate or prolonged metabolic stress.

Recent studies identified a number of miRNAs as a novel player in tightly controlling autophagy and maintaining cell viability as well as intracellular homeostasis [86, 87]. The polycistron miR-17~92 and its paralog miR-106b-25, two miRNA clusters frequently highly expressed in HCC [88, 89], were identified as regulators of autophagy by targeting p62, a multifunctional signal transducer modulating transportation of polyubiquitinated proteins for proteasome degradation [90]. Ectopic expression of these two clusters significantly promotes cell proliferation and differentiation capacity of myeloid progenitors. Moreover, several HCC-relevant miRNAs such as miR-182, miR-23b, miR-101, and miR-224 were shown to participate into the regulation of autophagy. Peng et al. demonstrated that miR-182 is significantly upregulated in prostate cancer cells in response to Atorvastatin, a putative inducer of autophagy in prostate cancer cells. Further experiments indicate that the proautophagic capacity of miR-182 is partially mediated by its downstream targets Bcl-2 and p21 [91]. By using radioresistant pancreatic cancer cell model, Wang et al. unveiled that overexpression of miR-23b attenuates radiation-mediated autophagy and sensitizes cancer cells to irradiation therapy through targeting Atg12 [92]. Tazawa et al. developed an engineered telomerase-specific oncolytic adenovirus with overexpression of tumor suppressive miR-7 and revealed that ectopic expression of miR-7 inhibits cell proliferation and triggers autophagy through a novel E2F1-miR-7-EGFR axis [93]. Very recently, Lan et al. uncovered a noncanonical pathway including miR-224 and autophagy in hepatitis B virus (HBV) related HCC patients [94]. Interestingly, mature miR-224 is preferentially localized into the autophagosome, where miR-224 undergoes degradation. However, in the HBV-associated HCC patients, due to unknown mechanism, low level of autophagy occurred and may partially account for the elevated expression of miR-224 in liver cancer [94]. Considering the important role of autophagy in cancer biology, attempts have been made to suppress tumor growth by miRNA-mediated inhibition of autophagy [95, 96]. However, further investigations are needed to establish the missing link for miRNAs and autophagy.

## 8. Conclusion and Perspectives

Currently, it has been well established that deregulation of miRNA expression significantly contributes to liver cancer progression. In most cases, miRNAs exert their function through induction translational inhibition and target mRNA degradation. Nevertheless, growing evidences suggest that they may possess other yet unknown functions such as behaving as transcription activator in gene regulation. Despite the fact that inspiring progress has been achieved in miRNA-mediated gene activation, many questions remain to be further elucidated. On the other hand, the identification of miRNA signatures aberrantly expressed in HCC paves the way to have a better understanding of the classification and assessment for diverse HCC subtypes in cancer patients. However, to achieve this goal, the specificity and accuracy of HCC-associated miRNA signatures need to be further verified with higher efficacy in the future studies.

The studies reviewed herein aim to highlight the overwhelming evidences regarding miRNA regulation of stemness characteristics of hepatic cancer stem cells, by modulating tumor-suppressive and oncogenic signaling pathways. The aforementioned new findings largely extend our understanding of HSCs regulation and shed light on developing novel therapeutic strategies to fight against chemotherapy-resistant HCC tumors. Given that the biogenesis of HSCs involved multiple steps including tumor initiation, epithelial mesenchymal transition, metastasis, and drug resistance to chemotherapy, miRNA-based therapy strategy that specifically attacks HSCs may launch novel firepower to the war against HCC. As individual miRNAs seem to distinctively modulate different aspect of stemness properties of HSCs (Figure 2), complete elimination of cancer stem cells in HCC tumors and the residual cancer cells may require the utility of multiple miRNAs in developing anti-HCC treatments.
